# Editorial

**Published:** 1880-10

**Authors:** 


### EDITORIAL DEPARTMENT.

This number completes the first volume of the Archives of Comparative Medicine and Surgery, and the first year of its existence. This journal was intended to supply a long-felt want, and to fill up an existing void in the department of science to which it is devoted. Notwithstanding the many perplexities and embarrassments which environed this periodical at its commencement, some of which are incidental to the inauguration of every new enterprise, the publishers are encouraged to persevere in their work; for they feel assured that their efforts have been appreciated by a discerning public. Scarcely a day passes without bringing evidence of this in the receipt of contributions and communications from some of the best thinkers and writers in this branch of science and practical literature; while our subscription list is steadily increasing by the addition to it of the names of readers which come to us voluntarily and without solicitation on our part.

We desire to have it distinctly understood that this Journal is not published in the interest of any individual, clique, school, or party, nor is it the mouthpiece of any society, but has been established purely in the cause of science. Its pages are open to all who will contribute anything new to the cause, and the editor will thank those who will send any communication (whether in the shape of an original essay or exact notes of clinical cases) that will help to throw new light on the important subjects to which the paper is devoted.

---

We are in receipt of a letter from Prof. James Law, of Ithaca, N. Y., calling our attention to a report made to the Commissioners of Agriculture by Dr. C. P. Lyman, and which was copied into the last number of the ARCHIVES. It is stated in this report that there were in the State of New York at that time, April 1880, five counties where the bovine lung plague was prevalent. This Prof. Law pronounces a mistake, and unjust to the

State officials, because at the time spoken of the cities of New York and Brooklyn were the only localities where the plague was known to exist, and the herds there would have been summarily disposed of had the requisite pecuniary aid been furnished by the authorities. We hasten to make this correction, as an error of this kind left on record without contradiction many soon become part of the history of this destructive epidemic, and may reflect on the manner in which those to whom the investigation and stamping out of this plague was committed executed their tasks. It is now generally conceded that these gentlemen were faithful and indefatigable in their efforts to carry out the purposes for which the commission was created. We can only regret that they were not more ably supported pecuniarily and otherwise. The loss which has occurred in this State alone from this plague already is doubtless tenfold greater than any expenditure asked for by this commission.

### VIVISECTION.

The July number of *Scribner's Monthly* contained an article "Does Vivisection Pay?" which we took occasion to criticise quite freely in the last number of the ARCHIVES. The writer of the article in question took ground in opposition to the practice. In the current number of the same publication we see an article on the "Value of Vivisection," the writer of which supports substantially our position. The readers of that periodical will thus hear both sides of this question. The writer of this last article is Dr. H. C. Wood, who shows himself entirely competent to deal with the subject. He shows very conclusively, that for the last twenty-five years great strides have been made in different branches of medical science; as, for example, in diseases of the nervous system, and also in the use of many remedial agents, medicinal drugs, ets., etc.

We may here add that experiments upon the lower animals have settled the question as to the communicability of many diseases from animals to man, and from man to animals. This latter fact is clearly shown in an article, by Dr. George Fleming, editor of the British "*Veterinary Journal*," in an article on Tuberculosis from a sanitary and pathological point of view. It is here shown conclusively by experiments performed by different investigators, that tubercles can be communicated by individuals of the bovine species to each other; also that these are communicable in the case of others of the lower animals, such as the dog, rabbit, etc. That they can be communicated by respiration and by inoculation, and as already well known, that they can be transmitted from the parent to the offspring. That they may be conveyed through the milk of tuberculous cows to hogs, and other animals fed upon it.

It has also been demonstrated that they may be communicated from animals to man, by inoculating. In proof of the above, he gives as authority, Gerlach, Gray, Epstein, Bromley, Toussaint, and others. To this we may add the testimony of Drs. Law, D. W. Voyles, Detmers, and D. E. Salmon veterinary surgeons, who, each for himself, and independently of the other instituted a series of experiments to ascertain the communicability of hog cholera, and bovine lung-plague

The results of these investigations will come home to any of our readers of average intelligence. We hope that our legislators will hereafter be deterred from interfering with these investigations, or the investigators who are by these experiments throwing so much light in the dark places of the laws of life.

---

We notice, in a commmunication published in the *Veterinary Journal*, that the Veterinary profession is becoming a political force. We are glad to make note of this fact, for it seems to us that through no other channel can substantial aid be had to support the cause of Veterinary science, than through our lawmakers. Veterinary science in Great Britain, as in this country, is languishing, not from the want of interest, but from the lack of funds to conduct the experimental researches, which are necessary to nourish from infancy to maturity this all-important branch of practical science. Agricultural colleges have received munificent appropriations. Literary colleges, and schools have also received public aid. Astronomical and meteorological observatories have all received aid from government on the ground of the public good; but this important branch has been practically neglected, both practice and investigation has been left in the hands of private individuals, who by their enthusiasm and love for these studies, have reacted upon the schools, while depending on their own resources mainly, supplemented to some extent by the necessarily small resources of their pupils. We hope, therefore, that the legislature of the State of New York will, at its next session, be fully awakened to a sense of its duty in this regard, and give to these schools some substantial aid in the way of appropriations, to enable them to carry on such investigations as may be necessary. We will simply refer to the scientific investigations carried on abroad and at home, and to the aceomplished men required to make them. These all result in good to the general public.

